# Improving spaCy dependency annotation and PoS tagging web service using independent NER services

**DOI:** 10.5808/GI.2019.17.2.e21

**Published:** 2019-06-24

**Authors:** Nico Colic, Fabio Rinaldi

**Affiliations:** 1 Institute of Computational Linguistics, University of Zurich, CH-8050 Zurich, Switzerland; 2 IDSIA, CH-6928 Manno, Switzerland; 3 Swiss Institute of Bioinformatics, Quartier Sorge-Bâtiment Amphipôle, CH-1015 Lausanne, Switzerland

**Keywords:** dependency parsing, named entity recognition, natural language processing

## Abstract

Dependency parsing is often used as a component in many text analysis pipelines. However, performance, especially in specialized domains, suffers from the presence of complex terminology. Our hypothesis is that including named entity annotations can improve the speed and quality of dependency parses. As part of BLAH5, we built a web service delivering improved dependency parses by taking into account named entity annotations obtained by third party services. Our evaluation shows improved results and better speed.

**Availability:** The code for our web service is publicly available (https://github.com/OntoGene/blah5).

## Introduction

Dependency parsing might be used as a component for tackling many text mining problems. It is often used as a feature to train machine learning algorithms or used for rule-based approaches for relation extraction [1,2]; and can be used to train better word embeddings [3]. However, as continued research and community efforts in the form of the CoNLL shared tasks, for example, show, dependency parsing in general is still not a problem solved completely [4]. Making a fast, reliable dependency parser readily available for the wider research community for further processing to build upon will help spur efforts in event and relation extraction.

As previous research has shown, providing named entity information to the dependency parser can improve the accuracy of the parses [5,6]. The reasoning is that dedicated named entity recognition (NER) tools perform much better in their specific domain, and by extracting named entities with higher accuracy will facilitate appropriate parsing tools that are not trained on biomedical data.

spaCy is a open-source natural language processing (NLP) library written in Python that performs tokenization, Part-of-Speech (PoS) tagging and dependency parsing. It is the fastest NLP parser available, and offers state-of-the-art accuracy [2,7].

Services such as PubDictionaries and OGER perform dictionary-based entity look up [8]. Other state-of-the-art taggers are the Jensen tagger [9] and TaggerOne [10].

**Fig. 1.**
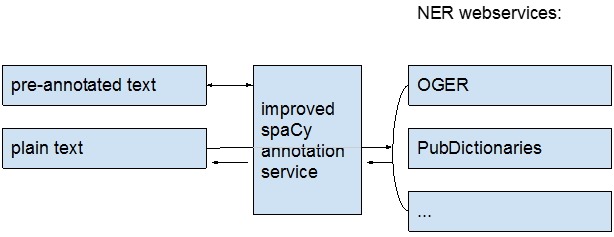
Architecture of our web service.

In this work, we present our contribution to BLAH5. We proposed to build a public web service that can be used by researchers and other services, such as PubAnnotation, to obtain improved dependency annotations, based upon integration with a NER service (Fig. 1). We report on its efficacy here.

## Related Work

The most recent extensive evaluation of existing dependency parsers has been performed by Choi et al. [11]. They evaluate 10 different off-the-shelf parsers for accuracy and speed; reporting labeled attachment scores (LAS) of 85% to 90%. While spaCy does not perform the most accurate in their evaluation, it performs fastest maintaining comparable accuracy. Similar scores are reported in the 2015 SemEval task [12].

Pletscher-Frankild and Jensen [9] note that in text mining literature, tools are often evaluated for accuracy only, omitting practical considerations such as speed and robustness.

Since the publication of the previously mentioned evaluation of dependency parsers [11], new machine learning-based parsers have been published, surpassing existing approaches according to the respective authors’ own evaluation [13,14]. Amongs them, spaCy was updated to employ a neural network to improve performance [7]. As Yang et al. [15] show, however, domain adaptation remains an issue.

## Methods

Our web service is implemented using Flask (http://flask.pocoo.org/), a Python library that facilitates the creating of web interfaces, and the aforementioned spaCy (https://spacy.io). spaCy’s structure is modular, and processing text happens by calling the respective modules. For our approach, we added a further processing step between “tokenization” and “dependency parsing,” during which we will recompute token segmentation taking into account named entities provided. Then we pass the adjusted token offsets to the dependency parsing module.

### Input format

**Fig. 2.**
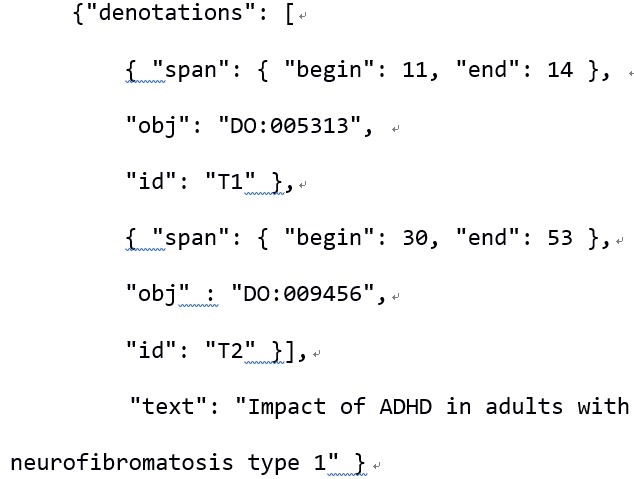
Example of input to our web service, in json format.

Input is provided either as plain text or as a JSON string. The latter may either contain just a text field, or additionally annotations in PubAnnotation's JSON format (http://www.pubannotation.org/docs/annotation-format/) (Fig. 2).

The input is then internally converted into a spaCy object. From there, spaCy is used to perform tokenization, PoS tagging and dependency parsing, taking into account existing annotations if available.

This is done by tokenizing the text provided, and iterating through the given annotations to form a new sequence of tokens. This new sequence keeps the NER annotations as individual tokens and does not break them down further. In case of conflicting and overlapping annotations, we use the leftmost longest match.

Using this new token sequence, a new spaCy representation object is created on which dependency parsing is performed.

If no annotations are provided manually, our web service will automatically call another service to automatically obtain named entity annotations. While this could be any service, it is currently set to using OGER.

### Output format

**Fig. 3.**
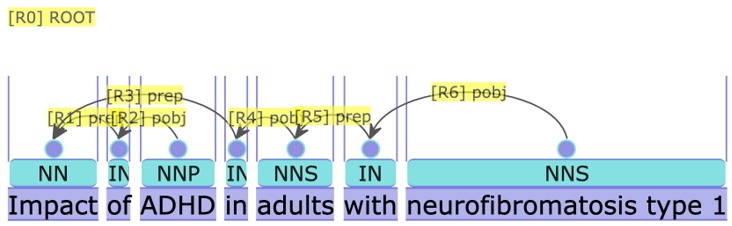
Visualizing the web service’s output in TextAE.

The resulting object is then converted back into JSON, and token offsets are computed. The output format is PubAnnotation JSON, and can directly be uploaded to PubAnnotation. This also allows to make use of PubAnnotation's annotation visualizer, *TextAE*, as seen in Fig. 3.

### Usage

The code for our web service is publicly available (https://github.com/OntoGene/blah5), and installation is straight-forward. For demonstration purposes, we are running an instance of the web service.

**Fig. 4.**
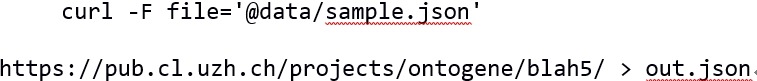
Example of complete query to the dependency annotation server: sample.json is the input file, out.json the output file, both in json format.

Improved dependency annotations can be obtained using curl or wget in the fashion shown in Fig. 4.

### Adaptation

The application can be run locally, provided the necessary Python libraries are available. To add other independent web services to provide NER information, refer to the documentation of the code.

## Results

We evaluated our service in two regards: speed and accuracy.

### Speed

In order to evaluate speed of our web service, we created a set of 10,000 randomly selected PubMed abstracts, (1) which we sent to the web service directly to obtain “default” dependency parses, and (2) which we first tagged for named entities using OGER, and then sent to the web service to obtain “improved” parses. Table 1 lists the time it took our tool to complete the dependency parsing step for both cases.

We attribute this decrease in processing time to the diminished complexity of computing the parses.

In a practical setting, however, our web service needs to recompute token offsets in order to correctly integrate NERs in the token stream.

While spaCy in the “default” case does this in a internally optimized fashion, our service currently is not optimized to the same degree, which in practice results in overall lower processing times. The integration of NERs still produces an advantage, as the generated parses avoid some errors that otherwise would have been generated (see below). In practice, when using the web service over the network, total processing time will be around 0.3 s per abstract.

### Accuracy

We expect our service to perform well in situations where multi-word named entities cause problems for the parser. Below we demonstrate two examples in which our service performs well in such situations.

**Fig. 5.**
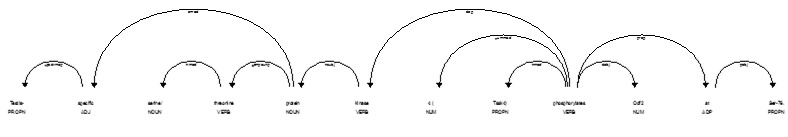
Default dependency parse for sentence *Testis-specific serine/threonine protein kinase 4 (Tssk4) phosphorylates Odf2 at Ser-76.*

**Fig. 6.**
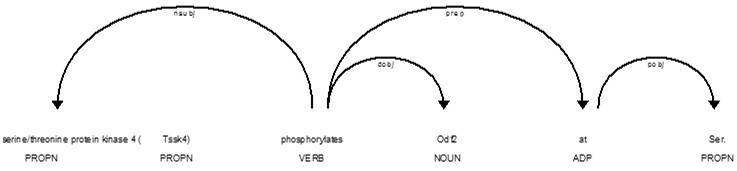
Improved dependency parse.

As Figs. 5 and 6 show, complex named entities are problematic for the dependency parser and tagger, causing it to tag “kinase” as a verb; when in fact it is part of the compound entity “Testis-specific serine/threonine protein kinase 4 (Tssk4).” This results in an incorrect parse tree.

**Fig. 7.**
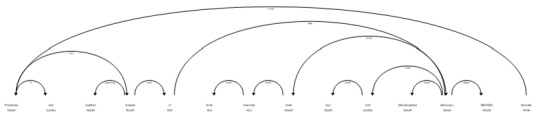
Default dependency parse for the sentence “Prevalence and mutation analysis of short/branched chain acyl-CoA dehydrogenase deficiency (SBCADD) detected.”

**Fig. 8.**
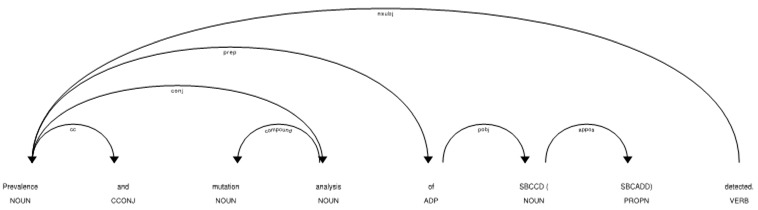
Improved dependency parse (“short/branched chain acyl-CoA dehydrogenase deficiency” abbreviated for display).

The second example of Figs. 7 and 8 shows the case where the parse is not technically wrong, but convoluted and useless to downstream tasks.

## Discussion

The examples shown above point to the efficacy of our web service. However, a more thorough evaluation, while useful, is difficult. This is mainly due to the fact that manual creation of parsing annotations is prohibitively expensive, and the fact that existing annotated corpora are annotated in various incompatible formats. For example, depending on the annotation schema, conjunctions can be represented in various ways, or relationships may be either undirected or have opposite directionality. Even though there are efforts to homogenize annotation schemes, such as Universal Dependencies [16], they are not yet widely adopted. This makes automatic comparison of results very difficult. However, since dependency parsing is not usually a goal in itself, but a component used for downstream tasks, an extrinsic evaluation could be carried out on such a downstream task such as relation extraction.

## Conclusion

We have presented an improved service for dependency parses, and show that it can deliver better parses for phrases containing complex tokens.

## Figures and Tables

**Table 1.** Time required to process a batch of 10,000 PubMed abstracts, in the default case (without prior named entity recognition) and with added named entity recognition

	*Default*	*Improved*
Total time (s)	1,018	931
Per abstract (s)	0.10	0.09

## References

[1] Nguyen TH, Grishman R (2015). Relation extraction: perspective from convolutional neural networks.

[2] Colic N (2016). Dependency parsing for relation extraction in biomedical literature.

[3] Levy O, Goldberg Y, Toutanova K, Wu H (2014). Dependency-based word embeddings.

[4] Zeman D, Hajic J, Popel M, Potthast M, Straka M, Ginter F, Zeman D, Hajic J, Popel M, Straka M, Nivre J, Ginter F (2018). CoNLL 2018 shared task: multilingual parsing from raw text to universal dependencies.

[5] Rinaldi F, Dowdall J, Hess M, Kaljurand K, Koitand M, Kahusk N, Melby A (2002). Terminology as knowledge in answer extraction.

[6] Lample G, Ballesteros M, Subramanian S, Kawakami K, Dyer C (2016). Neural architectures for named entity recognition. https://arxiv.org/abs/1603.01360.

[7] Honnibal M, Johnson M (2015). An improved non-monotonic transition system for dependency parsing.

[8] Furrer L, Jancso A, Colic N, Rinaldi F (2019). OGER++: hybrid multi-type entity recognition. J Cheminform.

[9] Pletscher-Frankild S, Jensen LJ (2019). Design, implementation, and operation of a rapid, robust named entity recognition web service. J Cheminform.

[10] Leaman R, Lu Z (2016). TaggerOne: joint named entity recognition and normalization with semi-Markov Models. Bioinformatics.

[11] Choi JD, Tetreault J, Stent A, Zong C, Strube M (2015). It depends: dependency parser comparison using a web-based evaluation tool.

[12] Oepen S, Kuhlmann M, Miyao Y, Zeman D, Cinkova S, Flickinger D, Nakov P, Zesch T, Cer D, Jurgens D (2015). SemEval 2015 task 18: broad-coverage semantic dependency parsing.

[13] Dozat T, Manning CD (2016). Deep biaffine attention for neural dependency parsing. https://arxiv.org/abs/1611.01734.

[14] Kiperwasser E, Goldberg Y (2016). Simple and accurate dependency parsing using bidirectional LSTM feature representations. Trans Assoc Comput Linguist.

[15] Yang H, Zhuang T, Zong C (2015). Domain adaptation for syntactic and semantic dependency parsing using deep belief networks. Trans Assoc Comput Linguist.

[16] Nivre J, de Marneffe MC, Ginter F, Goldberg Y, Hajic J, Manning CD, Calzolari N, Choukri K, Declerck T, Goggi S, Grobelnik M, Maegaard B (2016). Universal dependencies v1: a multilingual treebank collection.

